# Overexpression of miR-199a-5p decreases esophageal cancer cell proliferation through repression of mitogen-activated protein kinase kinase kinase-11 (MAP3K11)

**DOI:** 10.18632/oncotarget.6752

**Published:** 2015-12-24

**Authors:** Kimberly A. Byrnes, Pornima Phatak, Daniel Mansour, Lan Xiao, Tongtong Zou, Jaladanki N. Rao, Douglas J. Turner, Jian-Ying Wang, James M. Donahue

**Affiliations:** ^1^ Department of Surgery, Cell Biology Group, University of Maryland School of Medicine, Baltimore, MD 21201, U.S.A; ^2^ Baltimore Veterans Affairs Medical Center, Baltimore, MD 21201, U.S.A; ^3^ Department of Pathology, University of Maryland School of Medicine, Baltimore, MD 21201, U.S.A

**Keywords:** miR-199a-5p, MAP3K11, esophageal cancer, mRNA stability, proliferation

## Abstract

Studies examining the oncogenic or tumor suppressive functions of dysregulated microRNAs (miRs) in cancer cells may also identify novel miR targets, which can themselves serve as therapeutic targets. Using array analysis, we have previously determined that miR-199a-5p was the most downregulated miR in two esophageal cancer cell lines compared to esophageal epithelial cells. MiR-199a-5p is predicted to bind mitogen-activated protein kinase kinase kinase 11 (MAP3K11) mRNA with high affinity. In this study, we observed that MAP3K11 is markedly overexpressed in esophageal cancer cell lines. Forced expression of miR-199a-5p in these cells leads to a decrease in the mRNA and protein levels of MAP3K11, due to decreased MAP3K11 mRNA stability. A direct binding interaction between miR-199a-5p and MAP3K11 mRNA is demonstrated using biotin pull-down assays and heterologous luciferase reporter constructs and confirmed by mutational analysis. Finally, forced expression of miR-199a-5p decreases proliferation of esophageal cancer cells by inducing G2/M arrest. This effect is mediated, in part, by decreased transcription of cyclin D1, due to reduced MAP3K11-mediated phosphorylation of c-Jun. These findings suggest that miR-199a-5p acts as a tumor suppressor in esophageal cancer cells and that its downregulation contributes to enhanced cellular proliferation by targeting MAP3K11.

## INTRODUCTION

Esophageal cancer is now the 6^th^ leading cause of cancer-related death in the world [1]. In 2015, it is estimated that approximately 17,000 cases of esophageal cancer will be diagnosed in the United States, while 15,590 deaths will result from esophageal cancer [2]. Despite employing multimodality therapy consisting of chemotherapy, radiation, and surgery, overall 5-year survival is dismal at 18%. Given the rising prevalence of this deadly disease, increased efforts aimed at understanding the molecular mechanisms underlying the development and progression of esophageal cancer are urgently required. Such efforts are needed to develop predictive and prognostic biomarkers as well as to identify candidates for targeted therapies.

MicroRNAs (miRs) are now well recognized as critical post-transcriptional regulators of gene expression in cancer cells [3]. Individual miRs can modulate multiple biologic processes based on their ability to target several distinct transcripts [4]. Dysregulation of miR expression has been observed in multiple malignancies [5]. MiRs can act as both oncogenes and tumor suppressors by affecting critical processes such as cellular proliferation, sensitivity to chemotherapy-induced apoptosis, promotion of angiogenesis, invasiveness, and formation of metastases. Interestingly, the particular role played by an individual miR may differ depending on the specific malignant cell type.

Using miR array technology, several groups have compared miR expression in human esophageal cancer samples to matched normal controls. These studies have demonstrated distinctive patterns of miR expression in esophageal adenocarcinomas and squamous cell cancers that distinguish these malignancies from normal tissue [6–8]. Although numerous reports have detailed the targets of some miRs in esophageal cancer cell lines, currently a paucity of data exists regarding differences in miR expression patterns between esophageal cancer cell lines and esophageal epithelial cells. We have previously compared global miR expression in a human esophageal epithelial cell line (hESO) to the human esophageal squamous cell cancer lines TE7 and TE10 using array analysis [9]. When the 30 miRs with the greatest magnitude of differential expression between the cancer cells and epithelial cells were analyzed, 18 were found to share similar expression patterns in both TE7 and TE10 cells. Eleven were found to be markedly down-regulated in the cancer cells lines and 7 markedly upregulated compared to the hESO cells. miR-199a-5p was found to be the most down-regulated miR in both TE7 and TE10 cells compared to the hESO cells.

Based on miR target prediction programs, miR-199a-5p is predicted to bind mitogen-activated protein kinase kinase kinase 11 (MAP3K11) with high affinity. MAP3K11 is a critical component of the MAP kinase pathway which regulates several proteins involved in proliferation, apoptosis, migration, and invasiveness. Overexpression of MAP3K11 has been demonstrated in multiple malignancies, but no evidence exists on its expression levels in esophageal cancer. The goal of this study was to examine expression of MAP3K11 in esophageal cancer cell lines and to characterize the interaction between MAP3K11 mRNA and miR-199a-5p in these cells using functional and binding assays. In addition, we describe the phenotypic effects of modulating expression of miR-199a-5p in these cells.

## RESULTS

### miR-199a-5p and MAP3K11 exhibit dichotomous expression patterns in esophageal cancer cell lines

**Figure 1:**
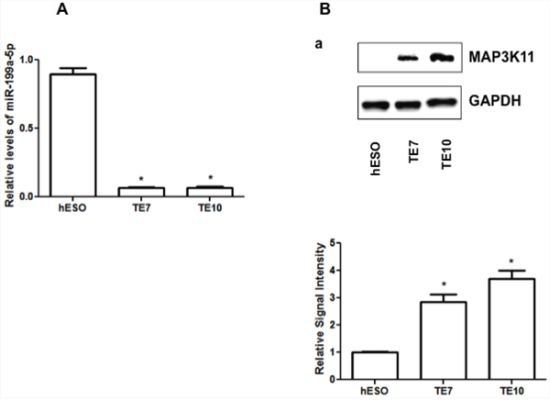
Baseline miR-199a-5p and MAP3K11 protein expression levels in human esophageal cell lines **A.** miR-199a-5p expression levels in hESO, TE7 and TE10 cells by q-PCR. **B.** Baseline protein expression levels of MAP3K11 in hESO, TE7 and TE10 cells. Representative experiment of three independent experiments. The adjacent bar diagram for relative protein signal intensity displays the mean signal intensity of three independent experiments. Signal intensity of target protein is determined and is normalized by signal intensity of GAPDH. Relative signal intensity is calculated compared to normal epithelial cells and is shown as bar diagram. Mean ± SD from three independent experiments. Asterisk, statistical significance based on two-tailed Student's *t* test. Signal intensity is determined using Bio-RAD image lab quantification software. Error bars represents ± S.D. and statistical significance based on a two-tailed Student's *t* test is indicated by *(*p* < 0.05).

In array analysis of global miR expression in human esophageal epithelial cells (hESO) and the human esophageal squamous cell cancer lines TE7 and TE10, miR-199a-5p was found to be the most down-regulated miR in both cancer cell lines [9]. These findings were confirmed by harvesting total RNA from all three cell lines and performing real-time PCR (q-PCR) analysis to measure miR-199a-5p levels. The data in Figure 1A show that miR-199a-5p levels are significantly decreased in TE7 and TE10 cells compared to hESO cells.

Based on a review of the Target Scan 6 and miRDB target prediction programs, MAP3K11 contains two potential high affinity binding sites for miR-199a-5p. We predicted that MAP3K11 levels should be high in the cancer cells lines if this interaction were biologically meaningful. In support of this hypothesis, we found that baseline levels of MAP3K11 are indeed elevated in TE7 and TE10 cells in comparison to hESO cells (Figure 1B).

### Modulating miR-199a-5p levels leads to alterations in MAP3K11 protein expression

**Figure 2:**
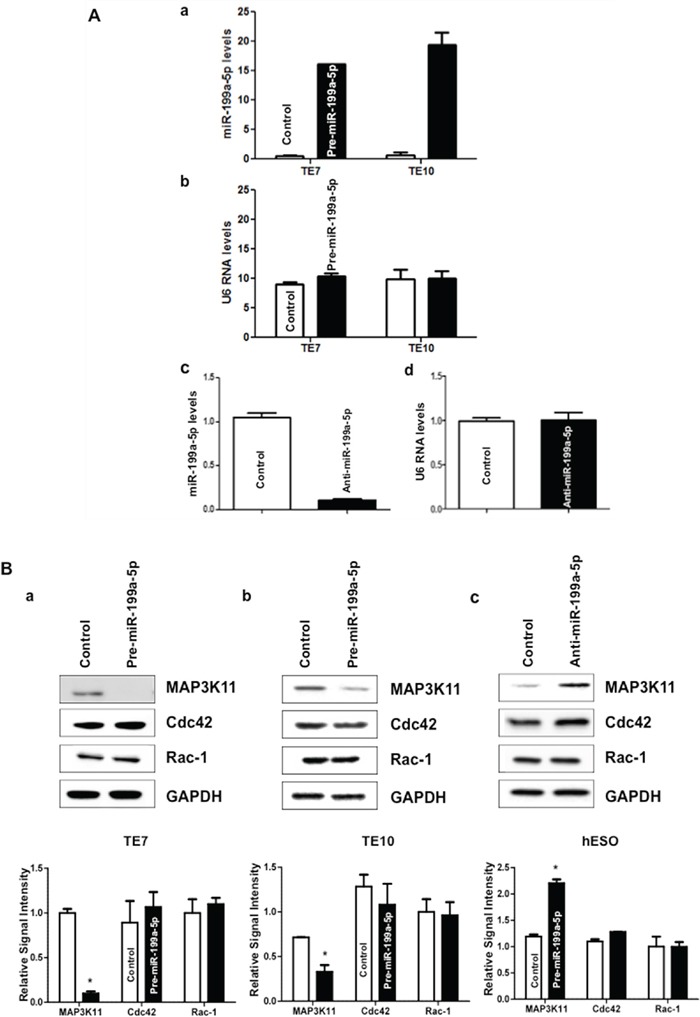
miR-199a-5p negatively regulates MAP3K11 expression in human esophageal cell lines **A.** Cells were transfected with control miR or **(a)** with 10 nM pre-miR-199a-5p (TE7 & TE10) or **(c)** with 25 nM anti-miR-199a-5p (hESO). Forty-eight hours post-transfection, levels of miR-199a-5p and U6 RNA **(b, d)** were measured by q-PCR. Values are mean ± SD from three independent sets of experiment in triplicate. **B.** In similar experiments, whole cell lysates were isolated and subjected to western blot analysis with indicated antibodies. Changes in MAP3K11, Cdc42, and Rac-1 protein expression after pre-miR-199a-5p transfection in **(a)** TE7 and **(b)** TE10 cells. **(c)** Changes in above mentioned protein expression after silencing miR-199a-5p in hESO cells. Representative immunoblots of three independent experiments in all the cell lines. The adjacent bar diagrams for relative protein signal intensity are the mean signal intensity of three separate immunoblots shown in a, b and c. Error bars represents ± S.D. and statistical significance based on a two-tailed Student's *t* test is indicated by *(*p* < 0.05).

Because basal levels of miR-199a-5p are low in TE7 and TE10 cells, transfection of pre-miR-199a-5p into these cells was performed in order to assess the effects on MAP3K11 expression. In reciprocal experiments, anti-miR-199a-5p was employed to reduce miR-199a-5p levels in hESO cells. As shown in Figure 2A, transfection efficiency of pre-miR-199a-5p was robust in both TE7 and TE10 cells (a). Similarly, transfection of anti-miR-199a-5p was very effective in reducing miR-199a-5p levels in hESO cells (c). Following successful transfection of pre-miR-199a-5p, MAP3K11 protein levels are markedly decreased in TE7 and TE10 cells (Figure 2B a/b). Of note, there was no effect on protein levels of Cdc42 and Rac-1, two important upstream regulators of MAP3K11. Conversely, MAP3K11 protein levels were increased compared to control-miR transfection in hESO cells following transfection of anti-miR-199a-5p (c). There was no change in either Cdc42 or Rac-1 expression following silencing of miR-199a-5p in hESO cells.

### miR-199a-5p reduces MAP3K11 mRNA stability

**Figure 3:**
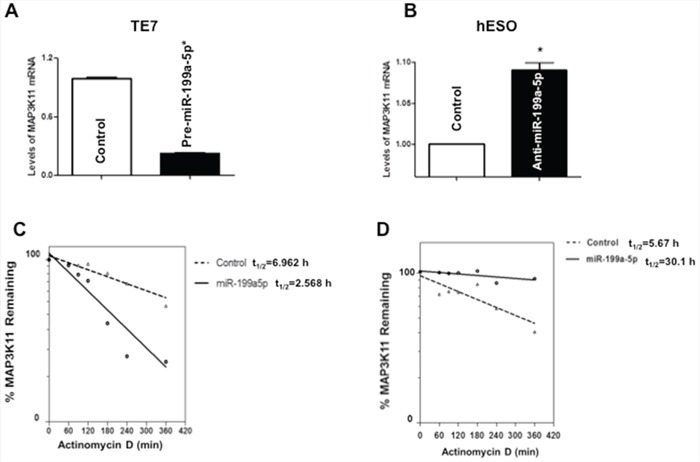
Effect of miR-199a-5p modulation on MAP3K11 mRNA levels **A.** Changes in levels of MAP3K11 mRNA in TE7 cells following transfection of pre-miR-199a-5p (10 nM) or control miR. **B.** Levels of MAP3K11 mRNA in hESO cells after transfection of anti-miR-199a-5p (25 nM) or control miR. In these experiments, 48 hours post-transfection, total RNA was extracted and levels of MAP3K11 were measured by q-PCR. Results represent the mean values of three biological and technical replicates. Error bars represents ± S.D. and statistical significance based on a two-tailed Student's *t* test is indicated by *(*p* < 0.05). **C.** Stability of MAP3K11 mRNA in TE7 cells following transfection of pre-miR-199a-5p (10 nM) or control miR. **D.** Stability of MAP3K11 mRNA in hESO cells after transfection of anti-miR-199a-5p (25 nM) or control miR. Total RNA was isolated at indicated time points after administration of Actinomycin D (4 μM) and the remaining levels of MAP3K11 mRNA were measured by q-PCR. Levels were normalized with GAPDH. The half-life was calculated from the first order equation t_1/2_ = ln2/k. Each point is the mean ± S.D. of three separate experiments.

To determine the mechanism by which miR-199a-5p affects MAP3K11 protein expression, levels of MAP3K11 mRNA were assessed following overexpression of pre-miR-199a-5p in TE7 cells, as well as following transfection of anti-miR-199a-5p in hESO cells. As seen in Figure 3A, transfection of pre-miR-199a-5p was associated with a decrease in MAP3K11 mRNA levels in TE7 cells. As anticipated, in hESO cells reduction of miR-199a-5p expression led to an increase in MAP3K11 mRNA levels (Figure 3B).

Figures 3C and 3D depict stability of MAP3K11 in TE7 and hESO cells following modulation of miR199a-5p levels. In these experiments, 48 hours following transfection, cells are exposed to 4 μM of Actinomycin D to prevent further transcription. Total cellular RNA is harvested at specified time points and levels of MAP3K11 mRNA are measured by q-PCR. As seen in Figure 3C, MAP3K11 is destabilized in TE7 cells following pre-miR-199a-5p transfection. The stability curve in Figure 3D demonstrates enhanced stability of MAP3K11 mRNA following silencing of miR-199a-5p in hESO cells.

### miR 199a-5p binds to MAP3K11 mRNA

**Figure 4:**
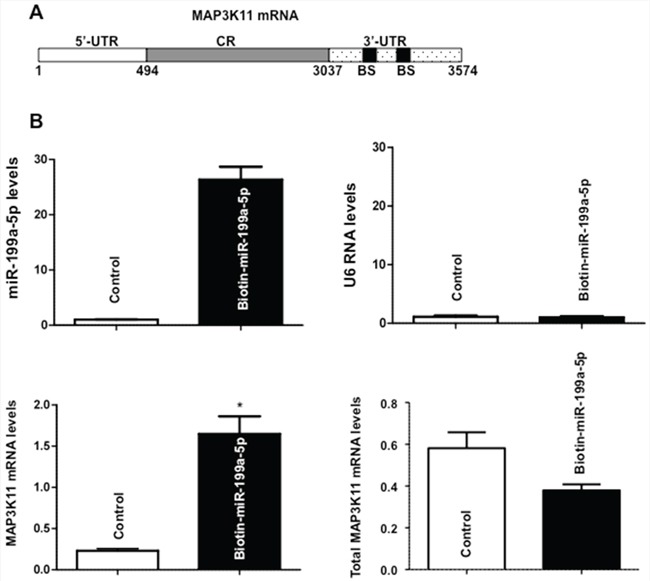
Association of miR-199a-5p with MAP3K11 mRNA **A.** Schematic representation of MAP3K11 mRNA. (BS) indicates predicted binding sites for miR-199a-5p. **B.** Levels of miR-199a-5p (Top panel, left) and U6 RNA (Top panel, right) 48 hours following transfection biotinylated-miR-199a-5p (50 nM) as measured by q-PCR analysis. Levels of MAP3K11 mRNA in the materials pulled down by biotin-miR-199a-5p (Bottom panel, left) and levels of total input mRNA (Bottom panel, right) measured by q-PCR. The enrichment of miR was calculated as follows: miR pull-down/control pull-down (X), miR input/control input (Y), Fold binding = X/Y. Representative bar diagram from three independent experiments. Each set of experiment was done in triplicate. Error bars represent mean ± S.D. and * stands for statistically significant based on two-tailed Student's *t* test where *p* < 0.05.

We next sought to determine whether miR-199a-5p directly interacted with MAP3K11 mRNA. As seen in Figure 4A, there are 2 predicted miR-199a-5p binding sites in the 3′ untranslated region (UTR) of MAP3K11 mRNA. As a first step in the binding analysis, biotin-labeled miR-199a-5p was transfected into TE7 cells and total cellular RNA was isolated and exposed to avidin-coated beads. RNA was harvested from the pull-down material and amplified with MAP3K11 probes by q-PCR. A biotin-labelled scrambled miR served as a control in these experiments. As seen in Figure 4B, the level of MAP3K11 mRNA was markedly elevated in the pull-down material isolated from TE7 cells following transfection with biotin-labeled miR-199a-5p compared to control.

**Figure 5:**
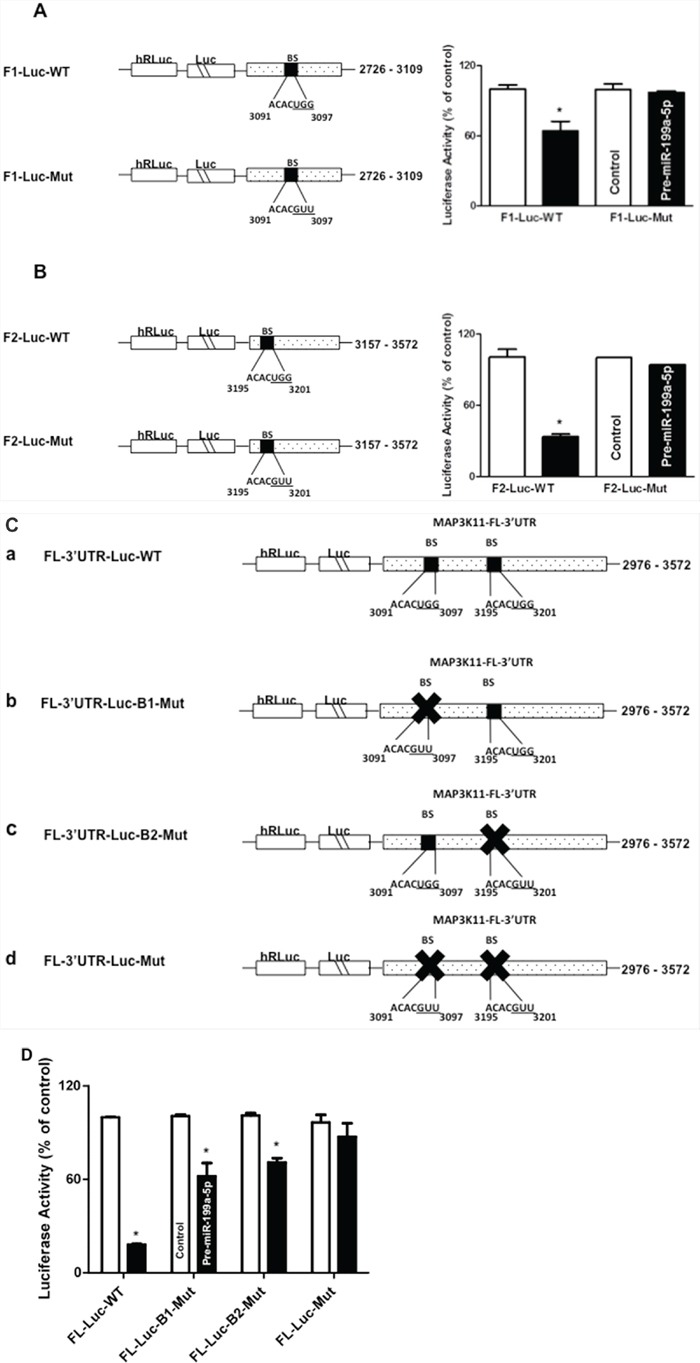
Contribution of potential miR-199a-5p binding sites in MAP3K11 mRNA **A.** and **B.** Schematic representations of MAP3K11 luciferase reporter constructs containing 3′UTR fragments with individual predicted miR-199a-5p binding sites, (A) F1 and (B) F2. In addition, the sequence of the miR-199a-5p potential binding sites in MAP3K11 3′UTR fragments F1 (A) and F2 (B) were mutated by substituting 3 bases (underlined). Adjacent bar diagrams depict luciferase activity in the specified wild-type (WT) and mutant (Mut) MAP3K11 reporter constructs (10ng) following co-transfection with pre-miR-199a-5p (10 nM) or control miR in TE7 cells for 36 hours. Firefly luciferase activities were normalized to Renilla luciferase activities and expressed as the mean of three independent experiments. All experiments were carried out in triplicate. Error bars represent mean ± S.D. and * represents statistically significant *p* < 0.05, *p* values based on two-tailed Student's *t* test. **C.** Schematic representation of constructs of different MAP3K11 luciferase reporter constructs containing the full length 3′UTR (FL-3′UTR). **(a)** The binding sequences of both of the miR-199a-5p potential binding sites are intact in the full-length MAP3K11 3′UTR wild-type (WT) fragment. **(b)** The binding sequence of miR-199a-5p potential binding site 1 was mutated by substituting 3 bases (underlined) while potential binding site 2 remained intact. **(c)** The binding sequence of miR-199a-5p potential binding site 2 mutated by substituting 3 bases (underlined) while potential binding site 1 remained intact. **(d)** Binding sequences of both the predicted miR-199a-5p binding sites in the MAP3K11 full-length 3′UTR were mutated. **D.** Luciferase activities of the mutated constructs (10 ng) were compared to wild type following co-transfection with pre-miR-199a-5p (10 nM) or control miR in TE7 cells for 36 hours. Firefly luciferase activities were normalized to Renilla luciferase activities and expressed as the mean of three independent experiments. All experiments were carried out in triplicate. Error bars represent mean ± S.D. and * represents statistically significant *p* < 0.05, *p* values based on two-tailed Student's *t* test.

In order to determine if both potential miR-199a-5p binding sites were functional, individual fragments of the MAP3K11 3′UTR (F1-Luc, F-2 Luc), each containing an individual predicted miR-199a-5p binding site were PCR amplified and separately sub-cloned into luciferase reporter vectors (Figures 5A and 5B). Following co-transfection with pre-miR-199a-5p or control miR, there was an approximately 39% reduction in luciferase activity with the F1-Luc construct and a 68.3% reduction in luciferase activity with the F-2 construct. Importantly, following mutation of 3 bases in the seed sequence binding region of the predicted miR-199a-5p binding site in each fragment, the reduction in luciferase activity following co-transfection with pre-miR-199a-5p was completely abrogated.

Finally, in order to better assess the relative contributions of each binding site, another series of luciferase reporter vectors were constructed (Figure 5C). The full length wild-type (FL WT) construct contained both intact binding sites. In B1-Mut, the first binding site was mutated while the second binding site was intact. Similarly, in B2-Mut, the second binding site was mutated, while the first binding site was intact. Finally, in the Mut construct, both binding sites were mutated. As seen in Figure 5D, mutation of either binding site, with the other binding site left intact, resulted in a partial recovery in luciferase activity following co-transfection with pre-miR-199a-5p compared to the FL WT construct. Co-transfection of the Mut construct, in which both binding sites were mutated, with pre-miR-199a-5p, resulted in near-complete abrogation of the decrement in luciferase activity, suggesting that both binding sites are functional.

### Overexpression of miR 199a-5p represses TE7 cell proliferation and induces G2/M cell cycle arrest, partially through inhibition of Cyclin D1 expression

**Figure 6:**
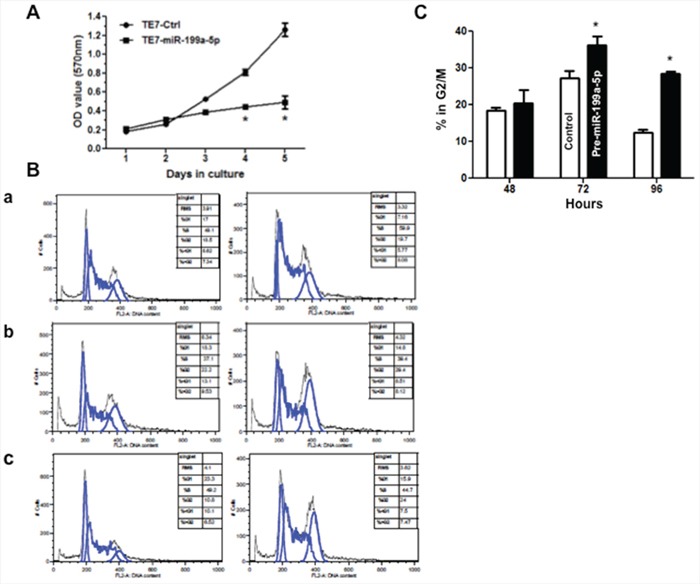
Overexpression of miR-199a-5p reduces proliferation and induces G2/M arrest in TE7 cells **A.** Following transfection with pre-miR-199a 5p (10 nM) or control miR, TE7 cells were harvested at the indicated time points, and were incubated with MTT for 4 hours at 37°C. The resultant growth curves are plotted based on the MTT assay results. The value shown is the mean of three independent experiments. Error bars represent mean ± S.D. and * represents statistically significant *p* < 0.05, *p* values based on two-tailed Student's *t* test. **B.** DNA content analysis. Following transfection with pre-miR-199a-5p (10 nM) or control miR, TE7 cells were harvested at various time points, fixed in 70% ethanol, and stained with propidium iodide. Cells were subsequently analyzed by flow-cytometry. Representative cell cycle histograms of control (left column) verses treated (right column) cells are shown at **(a)** 48 hours, **(b)** 72 hours, and **c.** 96 hours following transfection. Peaks corresponding to 2n (G1) and 4n (G2/M) DNA content are marked. **C.** Comparison of the percentage of cells in G2/M phase at various time points following transfection with pre-miR-199a-5p or control miR. Results expressed as the mean of three independent experiments. Error bars represent mean ± S.D. and * represents statistical significance, *p* < 0.05, *p* values based on two-tailed Student's *t* test.

Although multiple downstream effector pathways require activation through the MAP kinase signaling pathway, we chose to focus our evaluation of the effect of downregulation of MAP3K11 on cellular proliferation. As a first step in this analysis, as seen in Figure 6A, we observed a decrease in TE7 cellular proliferation following overexpression of miR-199a-5p, with a statistically significant decrease seen after 96 hours in culture. To better understand the mechanism underlying the observed reduction in proliferation, cell cycle analysis was performed. In keeping with the proliferation results, 96 hours following transfection with pre-miR-199a-5p, 24% of cells were in G2/M phase compared with 10.8% of cells following control transfection (*p* < .05).

**Figure 7:**
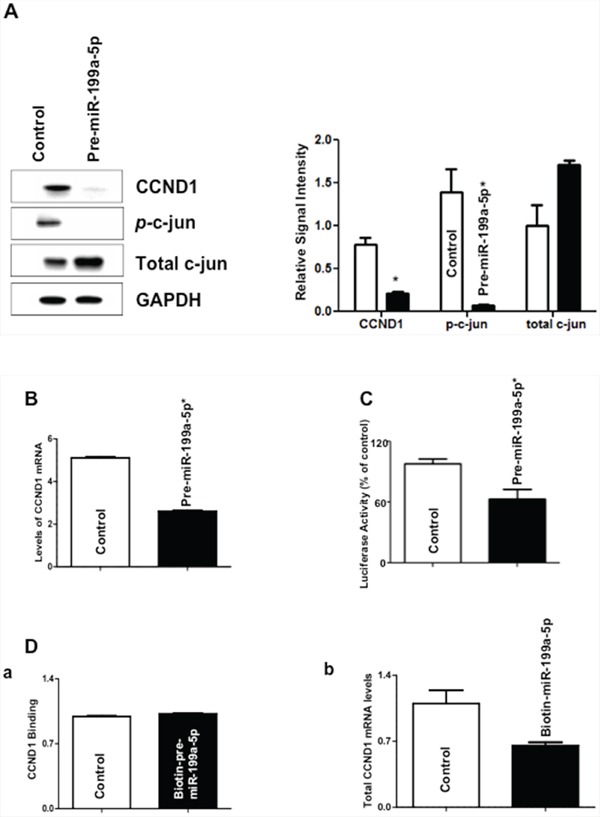
Effect of miR-199a-5p modulation on levels of c-jun and cyclin D1 (CCND1) **A.** Forty-eight hours following transfection with pre-miR-199a-5p (10 nM) or control miR, whole cell lysates were isolated and subjected to western blot analysis with indicated antibodies. Changes in levels of cyclin D1, phosphorylated c-Jun, and total c-Jun protein expression are depicted in the immunoblots. The adjacent bar diagrams for relative protein signal intensity are the mean signal intensity of three separate immunoblots. Signal intensity of target protein is determined and is normalized by signal intensity of GAPDH. Relative signal intensity is calculated compared to control and is shown as bar diagram. Results represent the mean ± SD from three independent experiments, and * represents statistical significance based on two-tailed Student's *t* test (*p* < 0.05). **B.** Changes in levels of cyclin D1 mRNA in TE7 cells following transfection of pre-miR-199a-5p (10 nM) or control miR. In these experiments, 48 hours post-transfection, total RNA was extracted and levels of cyclin d1 were measured by q-PCR. Results represent the mean values of three biological and technical replicates. Error bars represents ± S.D. and statistical significance based on a two-tailed Student's *t* test is indicated by * (*p* < 0.05). **C.** Luciferase activity in reporter constructs containing the cyclin D1 promoter (100 ng) following co-transfection with Renilla luciferase (10 ng) and either pre-miR-199a-5p (10 nM) or control miR in TE7 cells for 36 hours. Firefly luciferase activities were normalized to Renilla luciferase activities and expressed as the mean of three independent experiments. All experiments were carried out in triplicate. Error bars represent mean ± S.D. and * represents statistically significant *p* < 0.05, *p* values based on two-tailed Student's *t* test. **D. (a)** Levels of cyclin D1 mRNA in the pull down material 48 hours following transfection with biotinylated-miR-199a-5p (50 nM) or control miR. The enrichment of miR was calculated as follows: miR pull-down/control pull-down (X), miR input/control input (Y), Fold binding = X/Y. Representative bar diagram from three independent experiments, each set of experiment was done in triplicates. Error bars represent mean ±S.D. and * stands for statistically significant based on two-tailed Student's *t* test where *p* < 0.05. **(b)** Levels of cyclin D1 mRNA in the total input mRNA.

Because cyclin D1 is a key regulator of the G2/M transition and its transcription, is dependent, in part, by phosphorylation of c-Jun in a MAP3K11-dependent manner, we chose to investigate the effect of miR-199a-5p over-expression on cyclin D1 expression in TE7 cells [10–12]. As seen in Figure 7A, expression of cyclin D1 is markedly decreased following transfection with pre-miR-199a-5p. Furthermore, levels of phosphorylated c-Jun (Ser62) are markedly reduced, while total levels of c-Jun increased as would be expected from the reduction in phosphorylated c-Jun levels. In order to verify that the observed decrease in levels of cyclin D1 were due to decreased transcription, we next measured levels of cyclin D1 mRNA, which were decreased by approximately 50% following pre-miR-199a-5p transfection (Figure 7B). In addition, we found an approximately 40% reduction in cyclin D1 promoter activity following co-transfection of a luciferase reporter construct containing the cyclin D1 promoter with pre-miR-199a-5p compared to control (Figure 7C). Finally, because there is a predicted binding site for miR-199a-5p in the 3′ UTR of cyclin D1 mRNA, we investigated whether there was a direct interaction between miR-199a-5p and cyclin D1 mRNA. Figure 7D shows that there was no change in the level of cyclin D1 mRNA in the pull-down material isolated from TE7 cells following transfection with biotin-labeled miR-199a-5p compared to control.

## DISCUSSION

Our findings indicate that miR-199a-5p is markedly downregulated in esophageal squamous cancer cell lines compared to esophageal epithelial cells. We also demonstrate that miR-199a-5p regulates MAP3K11 expression in these esophageal cancer cells through a direct interaction with MAP3K11 mRNA. Forced expression of miR-199a-5p leads to a decrease in MAP3K11 mRNA and protein levels through decreased mRNA stability. Finally, the downregulation of MAP3K11 leads to decreased levels of phosphorylated c-Jun, ultimately resulting in decreased transcription of Cyclin D1. The resulting diminution in Cyclin D1 levels contributes to G2/M arrest and impaired cellular proliferation.

MiR-199a-5p, also referred to as miR-199-a in earlier literature, has been shown to be downregulated in multiple malignancies where it functions as a tumor suppressor by regulating such processes as cellular proliferation, sensitivity to chemotherapy-induced apoptosis, migration and invasiveness. The specific targets of miR-199a-5p vary across different malignancies. In prostate cancer cells, miR-199a-5p was shown to bind to the 3′UTR of GRP78, a major endoplasmic reticulum chaperone. Overexpression of miR-199a-5p led to decreased levels of GRP78, resulting in induction of apoptosis and increased sensitivity to the histone deacetylase inhibitor, trichostatin A [13]. In renal cell cancer (RCC) cells, overexpression of miR-199a-5p resulted in the downregulation of GSK-3β, a serine/threonine kinase involved in NFκB signaling. Proliferation was found to be significantly decreased in RCC cells following overexpression of miR-199a-5p [14]. In colorectal cancer cells, miR-199a-5p was found to interact with discoidin domain receptor 1 (DDR1), a receptor tyrosine kinase. Overexpression of miR-199a-5p led to decreased expression of DDR1 and resulted in decreased invasiveness and migratory ability of the transfected cells [15]. In addition, in ovarian cancer cells, miR-199a-5p has been shown to regulate expression of multiple oncogenic targets, suggesting that it may function as a master regulator in these cells. Restoration of miR-199a-5p expression in ovarian cancer cells has been shown to increase sensitivity to cisplatin, both through targeting mTOR and by targeting CD44, thereby reducing ovarian cancer stem cell levels [16, 17]. Finally, miR-199a-5p was also demonstrated to target IkappaB kinase-beta in ovarian cancer cells, resulting in increased sensitivity to TNF-α-induced apoptosis following forced expression of miR-199a-5p [18].

Although miR-199a-5p is generally found to be downregulated in cancer cells, it has been shown to be elevated in gastric adenocarcinomas [19–21]. Over-expression of miR-199a-5p has also been shown to be associated with advanced stage in these malignancies [22]. miR-199a-5p has been shown to function as an oncogene in gastric cancer cells partially through targeting klotho, which can suppress invasiveness and epithelial-mesenchymal transition [19]. Given the close relationship between gastric and esophageal adenocarcinomas, miR-199a-5p has also been shown to be elevated in esophageal adenocarcinomas and associated with increased stage [8, 23]. These studies are limited by relatively small numbers of patients. In addition, the difference in expression between normal and tumor tissue is variable, as is the number of patients in whom expression is found to be elevated. The fact that our studies were performed in esophageal squamous cancer cell lines also likely contributes to the discrepancy of our findings with these other reports, as the pattern of expression of miRs may vary significantly between esophageal adenocarcinomas and squamous cell cancers [6].

These data also illustrate the immense potential of miR expression profiling to identify not only individual miRs with important oncogenic functions within a particular cancer cell type, but also to identify new potential therapeutic targets based on identification of mRNAs regulated by the miRs. Observing the marked downregulation of miR-199a-5p in both the TE7 and TE10 cell lines enabled us to identify overexpression of MAP3K11 in these cells based on its predicted interaction with miR-199a-5p. MAP3K11 is an important component of the MAP kinase signaling pathways. In this system, an activated MAP kinase kinase kinase (MAP3K) phosphorylates and thus activates a MAPK kinase (MAP2K), which then phosphorylates a MAP kinase that activates a wide range of transcription factors that affect a broad array of cellular processes including differentiation, proliferation, migration, and apoptosis. MAP3K11, also known as mixed lineage kinase 3 (MLK3), is a member of the mixed lineage kinase subfamily of serine/threonine kinases. This family phosphorylates several MAP2Ks, which in turn may activate JNK and p38 [24–25]. In addition, MAP3K11 helps facilitate activation of B-Raf which stimulates ERK 1/2 signaling [26].

MAP3K11 has been shown to play a role in a variety of oncogenic processes in multiple malignancies. Silencing of MAP3K11 has been shown to decrease proliferation in colon cancer cells [26–27]. In lung cancer cells, MAP3K11 was found to be required for cell migration [28]. Similarly, MAP3K11 was found to be required for chemokine-induced invasiveness of breast cancer cells through its activation of paxillin [29]. In ovarian cancer cells, MAP3K11 was found to enhance invasiveness by regulating expression of multiple mixed metalloproteinases [30]. Importantly, overexpression of MAP3K11 induced malignant transformation of NIH3T3 cells through stimulating of the ERK pathway [31]. Based on these documented roles in proliferation, migration, and invasion, MAP3K11 has emerged as a potential therapeutic target. MLK inhibitors, such as CEP1347, have been developed primarily for treatment of neurodegenerative diseases [32]. The anti-tumor efficacy of these agents have not yet been tested in esophageal cancer patients.

Our findings contribute to an interesting developing paradigm in our understanding of the regulation of MAP3K11 by miRs, in which, although the same miR regulates MAP3K11 in different cell types, the phenotypic effects of the regulation may be in opposition. Levels of MAP3K11 were found to be increased in several primary melanoma samples. This was found to be associated with reduced levels of miR-125b. In melanoma cell lines, overexpression of miR-125b led to a decrease in MAP3K11 levels as well as decreased cell growth and invasiveness [33]. It is noteworthy that miR-125b is also significantly downregulated in TE7 and TE10 cells compared to hESO cells [9]. Although not directly evaluated in the current study, the downregulation of miR-125b may also contribute to the increased expression of MAP3K11 in these cells. In contradictory fashion, elevated levels of miR-125b were necessary to maintain murine pre-B cells in a transformed state. In these cells, although miR-125b was also found to negatively regulate MAP3K11, overexpression of MAP3K11 led to decreased growth and survival [34]. Similarly, our findings in esophageal cancer cells demonstrate that an increase in MAP3K11 levels, related to downregulation of miR-199a-5p, is associated with enhanced cellular proliferation. As was seen with miR-125b in murine pre-B cells, Song and colleagues have shown that miR-199a-5p was overexpressed in gastric cancer cells, associated with low levels of MAP3K11 [20]. As expected, silencing miR-199a-5p led to an increase in levels of MAP3K11. However, in opposition to our findings, the authors correlated the increase in MAP3K11 levels with the observed decreased proliferation of gastric cancer cells. Although there is limited data in these studies to show how MAP3K11 is affecting cellular proliferation and survival, it is conceivable that MAP3K11 may mediate very different cellular phenotypes depending on which signaling cascade it activates. For example, although in most cells, activation of MAP3K11 enhances cellular proliferation, it has been shown to induce cell death in neuronal cells. In these cells, activation of MAP3K11 activates JNK signaling, which in turn stimulates expression of pro-apoptotic proteins Fas Ligand and BH-3 only members of the BCl-2 family [35]. Future efforts aimed at understanding the functions of MAP3K11 in cancer cells will require detailed evaluation of the activated signaling pathways.

In this study, overexpression of miR-199a-5p with resulting decreased levels of MAP3K11 resulted in decreased cellular proliferation. When cell cycle analysis was performed, we found an increase in G2/M arrest associated with the observed reduction in proliferation. Several studies have described a role for cyclin D1 in progression through the G2 phase of the cell cycle [10–12]. We hypothesized that reduced MAP3K11 expression would result in decreased JNK mediated activation of c-Jun, a component of the AP-1 transcription factor, which enhances cyclin D1 transcription [36]. In support of this hypothesis, we observed decreased cyclin D1 levels, as well as decreased levels of phosphorylated c-Jun, despite no change in total c-Jun levels following overexpression of miR-199a-5p. Furthermore, we observed decreased cyclin D1 promoter activity following miR-199a-5p transfection. Importantly, as cyclin D1 mRNA does contain a potential binding site for miR-199a-5p, we found no evidence of direct binding of miR-199a-5p with cyclin D1 mRNA in biotin pull down experiments. Although overexpression of miR-199a-5p and downregulation of MAP3K11 may affect proliferation through other mechanisms not evaluated in this study, these data support an important role for cyclin D1 in mediating the observed decreased proliferation in esophageal cancer cells.

## MATERIALS AND METHODS

### Cell culture and reagents

The human esophageal squamous cancer cell lines TE7 and TE10 are kind gifts from Dr. Nishihira (Tohoku University, Sendai Japan). They were derived from esophageal squamous cell carcinomas from separate patients and are characterized in the Cell Resource Center for Biomedical Research at Tohoku University. These cell lines were cultured in RPMI media (Mediatech Inc, Herndon, VA) supplemented with 10% heat-inactivated FBS. hESO is an esophageal epithelial cell line derived from esophageal specimens harvested at the time of donor lung procurement. These cells were cultured in BEBM media (Lonza Corp, Walkersville, MD) supplemented with 20% heat-inactivated FBS and the BEGM bullet kit. All cells were maintained in a 37°C incubator with 5% CO2 humidified air. Pre-miR-199a-5p, anti-miR-199a-5p, and scrambled control-miRNA were purchased from Ambion (Austin, TX).

### miR Transfection

Transfections were carried out as previously described [9]. Cells were seeded in 6 cm plates at a density of 0.5–1 × 10^6^, a day prior to transfection. Pre-miR-199a-5p (10 nM), anti-199a-5p (25nM) or control miR was diluted in 500 μl Opti-MEM I (Invitrogen, Carlsbad, CA) containing 5 μl Lipofectamine RNAiMAX (Invitrogen, Carlsbad, CA). After 15 min incubation at room temperature, the complex was added to the cells in a final volume of 5 ml of fresh medium.

### Reverse transcription (RT) and quantitative real-time PCR (q-PCR) analyses

For all the RT and q-PCR experiments, total RNA was isolated from each sample using miRNeasy Mini Kit (Qiagen, Valencia, CA) according to manufacturer's manual and quantitated using NanoDrop1000 spectrometry (Thermo Scientific, Wilmington, DE). Equal amounts (1 μg) of total RNA were reverse transcribed using Oligo dt primer and AMV reverse transcriptase (Reverse Transcription System, Promega, Madison, WI). 10 ng of total RNA per 15 μl reaction was reverse transcribed using TaqMan miR reverse transcription kit with specific miR primers (Applied Biosystems, Foster City, CA). Q-PCR was performed in triplicate with specific (MAP3K11, miR-199a-5p, Cyclin-D1, U6 and GAPDH) TaqMan primers and probes (Applied Biosystems, Foster City, CA). Two μl cDNA were used in total 20 μl volume per reaction. Reactions were run on a STEP-ONE Plus Real–Time PCR System (Applied Biosystems, Foster City, CA) and cycling conditions were as follows: 2 min at 50°C, 10 min at 95°C, followed by 40 cycles of 15 s at 95°C and 1 min at 60°C. The threshold limit was set so that it intersected all the samples during the log-linear phase of amplification. The levels of GAPDH were used to normalize levels of MAP3K11 and cyclin D1 in q-PCR samples. For miR experiments, normalization was accomplished using small nuclear RNA U6.

### Immunoblotting

30 μg of protein from whole cell lysates was resolved on 10% SDS-PAGE gels (Bio-Rad Laboratories, Hercules, CA) and transferred onto PVDF membranes (GE Healthcare, Piscataway, NJ). After transfer, membranes were blocked in 5% nonfat milk in TBST and membranes were incubated with specific antibodies (overnight at 4°C) followed by horseradish peroxidase-conjugated anti-mouse or anti-rabbit (Santa Cruz, Dallas, TX) immunoglobulin for 1 hour at RT. Signal was detected by Chemiluminescence Reagent (PerkinElmer, Waltham, MA) and visualized by autoradiography. Anti-MAP3K11, anti-Cdc42, anti-c-Jun, anti-Rac-1, anti-cyclin D1 and anti-GAPDH were purchased from Santa Cruz Biotechnology (Dallas, TX). Anti-phosphorylated c-Jun (Ser62) II antibody was obtained from Cell Signaling Technology (Danvers, MA). Signal intensity was quantified using Image Lab quantification software (Bio-Rad, Hercules, CA).

### Bioinformatics

Two software programs, Target Scan 6.0 (http://www.targetscan.org/) and miRDB (http://mirdb.org/miRDB/), were used to predict the potential target genes of miR-199a-5p.

### mRNA stability

mRNA stability assays were performed as previously reported [37] and calculated using a nonlinear regression curve. Twenty-four hours following transfection as described above, medium containing Actinomycin D (Sigma–Aldrich, St. Louis, MO) at a final concentration of 4 μM was added for specified time points. Total RNA was isolated from each sample and qRT-PCR was performed in triplicate as described above.

### Biotin-labeled pull-down assays

Biotinylated miR-199a-5p (Dharmacon, Lafayette, CO) pull-down assay with target mRNAs was performed as described earlier [38–39]. Briefly, 1 × 10^6^ TE7 cells were seeded in 10 cm plate in duplicate a day before transfection. Next day, control miR or 3′ biotin-labeled miR-199a-5p (5′CCCAGUGUUCAGACUACCUGUUC 3′Bi) was transfected was transfected at a final concentration of 10 nM. After 48 hours, whole cell lysates were harvested. Simultaneously, Streptavidin-Dyna beads (Dyna beads M-280 Streptavidin, #11205D, Invitrogen, 50 μl each sample) were coated with 10 μl per sample yeast tRNA (stock 10 mg/ml Ambion, Austin, TX) and incubated with rotation at 4°C for 2 hrs. Then beads were washed with 500 μl lysis buffer and resuspended in 50 μl lysis buffer. Sample lysates were mixed with pre-coated beads (50 μl per sample) and incubated overnight at 4°C on a rotator. Beads were then pelleted down next day to remove unbound materials at 4°C for 1 minute, 5K rpm and washed five times with 500 μl ice cold lysis buffer. To isolate the RNA, 750 μl of TRIzol (Invitrogen, Carlsbad, CA) and 250 μl nuclease free water was added to both input and pulldown samples. Tubes were mixed well and kept in −20°C for 2 hrs. RNA was then precipitated using standard chloroform-isopropanol method and then subjected to q-PCR as explained above.

### Cell proliferation studies

TE7 cells were plated into 96-well plates at a density of 2 × 10^3^ cells/well in replicates of six. After 1, 2, 3, 4 and 5 days following transfection with pre-miR-199a-5p (10nM), cells were incubated with 3-(4–5-dimehtylthiazol-2-yl)-2,5-diphenyltetrazolium bromide (MTT, Roche, Mannheim, Germany) for ~4 h at 37°C. Subsequently, the supernatant was replaced with dimethyl sulphoxide to dissolve formazan crystal residues. A spectrophotometer was used to measure corresponding optical densities (absorbance at 570 nm). Each experiment was repeated at least thrice.

### Cell cycle analysis

TE7 cells were transfected with control miR or pre-miR-199a-5p for 48, 72, or 96 hrs. Following transfection, cell cycle analysis was performed as previously described [40]. Briefly, cells were harvested and washed twice with ice-cold phosphate-buffered saline (PBS) and fixed in 70% ice-cold ethanol. Cells were then resuspended in PBS containing RNase and propidium iodide (Sigma, St. Louis, MO) for 15 min at 37°C. Samples were run for DNA content using FACScan Flow Cytometer (BD Biosciences, San Jose, CA) and analyzed using FloJo v.8.8.7 software.

### Luciferase reporter assays

For MAP3K11 (NM_002419), individual luciferase reporter constructs were generated that contained either the full-length 3′ UTR, or one of 2 separate 3′UTR fragments. The inserts were amplified by PCR and individual fragments were subcloned into NheI and SalI (New England Bio Labs, Ipswich, MA) and digested pmirGLO Dual-Luciferase miRNA target expression vector (Promega Madison, WI). The constructs containing mutations at the seed region of potential binding sites were generated using site directed mutagenesis kit (Agilent Technologies, Santa Clara, CA). All primer sequences used to create these constructs are listed in Table 1. Restriction enzyme digestion and DNA sequencing confirmed the orientation and sequence of the constructs. For luciferase activity assay, 1 × 10^5^ TE7 cells/well were plated onto 12-well cell culture plates and co-transfected with luciferase reporter constructs (10ng) and pre-miR-199a-5p (10 nM) using Lipofectamine 2000 (Invitrogen, Carlsbad, CA) for 36 hours. Luciferase activity was measured using Dual Luciferase Reporter Assay kit (Promega, Madison, WI) according to the manufacturer's instructions. Levels of firefly luciferase activity were normalized to Renilla luciferase activity.

**Table 1:** Primer sequences for MAP3K11 luciferase reporters

Name	Sequence	Region	Cutting Site
pmirGLO-F1-Fwd	**GCTAGC**ACATCACGCTCTGCTCCTG	2726–3109	Nhe I
pmirGLO-F1-Rev	**GTCGAC**CCAGCTCCTGTTCCAGTGTA	2726–3109	Sal I
pmirGLO-F2-Fwd	**GCTAGC**TTGGGGGTCAGGAACACTAC	3157–3572	Nhe I
pmirGLO-F2-Rev	**GTCGAC**GTGGGGAGACAGCTTTTGAG	3157–3572	Sal I
pmirGLO-FL-Fwd	**GCTAGC**AGGGCACAGACCAAAGACAT	2976–3572	Nhe I
pmirGLO-FL-Rev	**GTCGAC**GTGGGGAGACAGCTTTTGAG	2976–3572	Sal I
pmirGLO-Mut-F1-Fwd	GGCTGACCCAGCTCCTGTTAACGTGTATGCTGTGACTCCTC	2726–3109	N/A
pmirGLO-Mut-F1-Rev	GAGGAGTCACAGCATACACGTTAACAGGAGCTGGGTCAGCC	2726–3109	N/A
pmirGLO-Mut-F2-Fwd	CAGGAAGCCTTCACACGTTAAGGGGGACCTGCGCC	3157–3572	N/A
pmirGLO-Mut-F2-Rev	GGCGCAGGTCCCCCTTAACGTGTGAAGGCTTCCTG	3157–3572	N/A

### Promoter activity

1748 human cyclin D1 promoter pGL3Basic was a gift from Frank McCormick (Addgene plasmid # 32726) and promoter activity was measured as described [41]. Briefly, 1 × 10^5^ TE7 cells/well were plated onto 12-well cell culture plates and co-transfected with reporter constructs containing the cyclin D1 promoter (100ng) and either pre-miR-199a-5p (10nM) or control miR in TE7 cells for 36 hours. 10 ng pRL-TK (Promega, Madison, WI) Renilla luciferase was co-transfected in each sample as an internal control for transfection efficiency. Experiments were performed in triplicate from independent cell cultures.

### Statistical analysis

Results are expressed as the means ± SDEV from three to six replicates for each set of experiment. Data derived from multiple determinations were subjected to Student's *t* test and *p* values < 0.05 were considered statistically significant.
